# Identification of Abiotic Stress Protein Biomarkers by Proteomic Screening of Crop Cultivar Diversity

**DOI:** 10.3390/proteomes4030026

**Published:** 2016-09-08

**Authors:** Bronwyn J. Barkla

**Affiliations:** Southern Cross Plant Sciences, Southern Cross University, Lismore 2481, NSW, Australia; Bronwyn.Barkla@scu.edu.au; Tel.: +612-6620-3159

**Keywords:** proteomics, biomarkers, abiotic stress, genetic diversity, crop improvement

## Abstract

Modern day agriculture practice is narrowing the genetic diversity in our food supply. This may compromise the ability to obtain high yield under extreme climactic conditions, threatening food security for a rapidly growing world population. To identify genetic diversity, tolerance mechanisms of cultivars, landraces and wild relatives of major crops can be identified and ultimately exploited for yield improvement. Quantitative proteomics allows for the identification of proteins that may contribute to tolerance mechanisms by directly comparing protein abundance under stress conditions between genotypes differing in their stress responses. In this review, a summary is provided of the data accumulated from quantitative proteomic comparisons of crop genotypes/cultivars which present different stress tolerance responses when exposed to various abiotic stress conditions, including drought, salinity, high/low temperature, nutrient deficiency and UV-B irradiation. This field of research aims to identify molecular features that can be developed as biomarkers for crop improvement, however without accurate phenotyping, careful experimental design, statistical robustness and appropriate biomarker validation and verification it will be challenging to deliver what is promised.

## 1. Introduction

It is estimated that there are 352,000 documented flowering plants in the world and anywhere from 30,000 to 80,000 of these contain parts that are in some form suitable for human consumption [1]. Historically, however, only about 7000 plants have actually been used for food [2], and since the industrial revolution, the exploitation of plant species diversity has progressively decreased. Modern agriculture has become reliant on fewer and fewer crop varieties for intensive farming practices, with today only about 150 species actively cultivated [3], and less than a dozen providing 80% of our caloric intake [4]. This practice, tending towards monoculture, raises serious food security issues. As we lose or disregard species diversity, the major crops that we increasingly rely on become vulnerable to environmental changes including both abiotic and biotic stress factors, as the genetic variation for stress tolerance is rather limited in domesticated gene pools which have a very narrow genetic base [3,5]. Genealogical analysis of the genetic diversity of Russian winter *Triticum aestivum* L. wheat underlines this genetic erosion, with 96% of all winter wheat varieties cultivated attributed to being descendants of only two cultivars, leaving it highly vulnerable [6]. The top three global staple crops, corn, wheat and rice, which make up more than 60% of the worldwide food production [5], are generally highly susceptible to stress at various stages of development. Unfavourable environmental conditions result in unacceptable decreases in quality and yield, affecting global annual agricultural production. 

As examples, in 1982, 1994 and 2002, unusually high temperatures, combined with low rainfall across southern Australia caused major crop failure, with less than half the normal wheat production attained [7]. More recently, in 2012, US corn production dropped 13 % as compared with previous years due to devastating drought, despite planting the largest acreage in a 75 year history (Crop Production 2012 Summary, USDA/NASS; www.nass.usda.gov). In general, adverse environmental conditions have been estimated to have lowered wheat and corn yields by roughly 6% and 4%, respectively, over a period of 30 years [8].

There is much interest to genetically improve crop plant stress tolerance. Global climate change and extreme weather conditions have resulted in greater temperature fluctuations, and drastically altered rainfall patterns; resulting in drought and/or flooding which in turn influences soil salinity. The cost of inorganic nitrogen is predicted to rise due to depletion of supplies and increased demand, necessitating breeding for better nitrogen use efficiency in crops. Furthermore, as the land available for agriculture becomes limiting, farmers are compelled to make use of marginal, poor quality soils, which can contain unfavourable levels of nutrients [9].

Much of our knowledge on abiotic stress tolerance has come from studies on the model plant, *Arabidopsis thaliana*, due to the early sequencing of the genome and policies which directed significant amounts of research funds to work on this plant [10]. It was believed that by adopting a single model species, the plant research community would make discoveries faster and test hypotheses more rapidly [11]. The availability of a large number of natural accessions of Arabidopsis adapted to varying environments was also an attractive feature of this model [12]. While Arabidopsis research has advanced plant functional genomics to a large degree, it is not the ideal model for all crop research, as it is a dicotyledonous weed while the three major crops are all monocots. Moreover, it does not produce fruit, and therefore, specific issues may not be addressed adequately by focusing solely on a single plant. Recently there has been a dramatic improvement in genomic technologies, enabling the sequencing of reference genomes for a wide variety of plants, including many crops (reviewed in [13]), facilitating direct research on the plant of interest and additionally providing access to the genetic diversity available within a species.

One approach that is gaining widespread interest in the community which emphasizes the utility of these newly obtained crop genomic resources is the exploitation of cultivars, landraces and wild relatives of major crops which are naturally more stress tolerant. These can be compared at the level of either the transcriptome or proteome to identify potential sources of tolerance for variety improvement. This approach has been not only employed for the major crops but also minor, less utilized crops have also been studied in this manner. In this particular review, the use of quantitative proteomics to understand differences in tolerance mechanisms within crop cultivars and identify possible abiotic stress markers by genotype comparison have been addressed and the data have been summarized below with respect to crop and stress type. Moreover, the relevant experimental design, including plant age, treatment type and length, tissue, extraction method and quantitative approach used in each case is listed in Table 1. 

## 2. Differential Proteomics to Identify Genotype Differences in Abiotic Stress Tolerance in Crops

### 2.1. Corn

#### 2.1.1. Water Deficit, Dehydration and Drought

The impact of drought on protein abundance in different corn cultivars has been addressed at the seedling state by two groups. Benešová et al. [14] studied drought-induced changes in the leaf proteome of two maize genotypes with contrasting sensitivity to dehydration brought about by withholding water, using both iTRAQ and 2DGE approaches. Results of iTRAQ analysis identified 220 proteins whose levels changed due to drought stress in at least one genotype by at least 2-fold, with 106 of these differentially expressed between genotypes. Not only did the drought tolerant cultivar, CE704, show upregulation of a much larger number of proteins than the drought-sensitive 2023, but many of these were also actually down-regulated in 2023. Of particular note were the upregulation of protective/detoxification associated proteins in CE704, including the antioxidant enzymes ascorbate peroxidase, superoxide dismutase and to a lesser extent glutathione reductase and catalase, as well as proteins involved in translation such as EF-TuM, a mitochondrial translation elongation factor, and eIF3, a translation initiation factor, in the drought-tolerant genotype.

In a similar study, Riccardi et al. [15] analysed protein changes in response to progressive water deficit in total protein from maize seedling leaf blades from two different genetically distinct lines (Io—drought-tolerant, Lc—drought-sensitive) and compared these to the hybrid of the two. Of the 78 drought-responsive proteins detected by 2DGE, 38 of these showed differential behaviour between the three genotypes. However, only a subset of those were sequenced. One protein, an ABA/water-stress/ripening related protein (ASR-protein), was found exclusively induced in the Io tolerant genotype, while malate dehydrogenase was found exclusively in the Lc genotype. The ASR protein has been described as a drought-responsive protein [47], but its function remains unknown. Riccardi also investigated the proteome of the elongation zone of the leaf blade in the same genotypes over multiple time points [48]. Many proteins identified in their earlier study were identified at one or more time points in this study. Increases in ABA45 and OSR40 were linked to differences in ABA accumulation between the genotypes and ASR1 was identified in both studies as a drought responsive protein [48].

#### 2.1.2. UV-Irradiation

Casati et al. [16] surveyed responses of maize landraces differing in their tolerance to UV-B radiation using Differential in Gel Electrophoresis (DIGE) of leaf total protein. Responsive protein profiles of a line deficient in red anthocyanin pigments (W23) due to mutation in chalcone synthase was compared with that of two high altitude maize landraces (Cacahuacintle and Confite Puneño). These high altitude landraces showed improved UV-B tolerance due to naturally elevated levels of UV-B radiation-absorbing flavones as well as reduced expression of stress related proteins. The study identified 53 proteins that were differentially regulated by UV-B radiation in W23 but showed constitutively high expression in at least one of the high altitude adapted landraces. This was narrowed down to ten proteins that showed increases in abundance in both the high altitude lines as compared to W23 and included, ribosome recycling factor, ferridoxin, actin PEPc, translation elongation factor Tu, 26S proteasome regulatory protein, hydroxyl pyruvate reductase, ADP-glucose pyrophosphorylase small subunit, succinyl-CoA synthase beta subunit and glutamate-1-semialdehyde mutase. The proteins identified had diverse biological function, which implied that enhanced UV-B tolerance of the high altitude lines was due to multiple unrelated factors. From this list, none of the expected ROS scavenging enzymes were identified as differentially responsive, but recent views hold that widespread, oxidative damage is rare under natural UV-B levels [49].

### 2.2. Wheat

#### 2.2.1. Drought

Quantitative proteomics using iTRAQ technology was used to monitor protein changes in total protein extracts from leaves of adult plants in response to cyclic drought conditions in three wheat cultivars differing in tolerance based on grain yield; RAC875 (drought tolerant), Excalibur (drought-tolerant), and Kukri (drought-intolerant) [17]. Cyclic drought was applied by withholding water until the least tolerant line wilted followed by rewatering and then repeating this treatment. While all three cultivars showed increases in proteins involved in oxidative stress metabolism, both drought-tolerant cultivars showed co-ordinated decreases in Calvin cycle enzymes after long-term water deficit. However, in general, the two drought-tolerant cultivars showed few similarities in their protein response, which was attributed to their phenotypic differences in maintaining grain yield when subjected to stress [17] suggesting distinct drought-tolerant mechanisms were employed in each species.

Using a similar iTRAQ-based quantitative proteomics approach but employing the wheat varieties Opata M85, an elite drought-sensitive cultivar and Nesser, a drought-tolerant cultivar, Alverez et al. [18], targeted differences in the seedling root proteome response to ABA. Both ABA-responsive and cultivar-specific protein changes were identified. Major differences included proteins involved in translation that were upregulated by ABA in Nesser but down regulated in Opata, as were heat shock proteins. Other proteins involved in signalling and transport, including P-ATPase, G-proteins, potassium channel, and calnexin were also selectively increased in the drought-sensitive cultivar by ABA [18]. Using validation by PCR most protein changes corresponded to gene expression changes for a number of selected candidates.

In another study, Hao et al. [19] compared a drought-tolerant wheat cultivar Hanxuan 10 to a drought-sensitive cultivar Chinese Spring under PEG induced drought stress and recovery. 2D-PAGE identified 16% of the proteins as being differentially expressed between the two cultivars in isolated total protein extracts from seedling roots, leaves and an intermediate section between the two. In contrast to results reported by Alvarez et al. [18], many stress-related heat shock protein chaperone family members were shown to be upregulated in the drought-tolerant cultivar and there appeared to be an increase in the phosphorylation level of these proteins. 

#### 2.2.2. Salinity

In one of the few studies that focuses on cultivar-specific protein changes restricted to a subcellular compartment, Jacoby et al. [20] analysed the mitochondrial role in salinity tolerance by comparing the salinity responses of bread wheat (CS) against a salt-tolerant amphiploid AMP (wheat × *Lophopyrum elongatum*); analysing samples from both shoots and roots. They identified a large number of mitochondrial proteins as responsive to salinity in both genotypes, including enzymes involved in reactive oxygen species detoxification. Two enzymes involved in one carbon metabolism, glycine decarboxylase, and serine hydroxymethyltransferase were specifically and significantly increased in abundance in the tolerant cultivar. These enzymes, working in tandem, are responsible for the essential primary metabolism reaction which involves the interconversion of glycine and serine using tetrahydrofolate as a cofactor, serving as the hub of all carbon metabolism in the plant [50]. Independent of stress, cultivar specific differences in protein composition of the mitochondria were also identified, with altered abundance of manganese superoxide dismutase, serine hydroxymethyltransferase, aconitase, malate dehydrogenase, and β-cyanoalanine synthase between the genotypes. It was suggested that these proteins could contribute to the higher salt-tolerance of AMP and serve as molecular markers for breeding salt-tolerant wheat varieties [20].

The independent effects of both drought and salinity stress on seedlings of another bread wheat cultivar cv. Jinan 177 were compared to the somatic hybrid stress tolerant wheat cv. Shanrong No. 3 (SR3) using 2DGE [22]. While the majority of both the root and leaf stress-responsive differentially abundant proteins were shared, whether the imposed stress was drought or salinity, there were genotype, stress, and tissue specific differences. Overall, salt-stress resulted in more changes in protein abundance than did drought stress. A number of these proteins were identified, however, the majority had only a single matched peptide and multiple spots corresponded to the same protein. The functions of these proteins were varied and included the alpha subunit of the F1-ATPase, and Rubisco large subunit [22].

#### 2.2.3. Salinity, Drought, Heat, or Cold

In another wheat study, Kamal et al. [21] contrasted four wheat cultivars, two Chinese cultivars (China-108 and Yennon-78) and two Japanese cultivars (Norin-61 and Kantou-107), with unknown stress phenotypes, to identify grain stress-inducible soluble proteins. All four cultivars showed the common induction of 11 proteins under the various stress regimes, several of which have known roles in stress responses including the ROS scavenging enzyme ascorbate peroxidase, as well as a truncated cold acclimation protein, and a salt-tolerant protein, both with unknown functions. Both Japanese cultivars showed the highest number of unique stress-inducible proteins with 24 and 19 uniquely responsive proteins identified in Norin-61 and Kantou 107, respectively, compared to only one (China-108) and four (Yeonnon-78) uniquely expressed proteins in the Chinese cultivars. Genotype differences did not show specific enrichment of proteins involved in specific cellular processes but rather proteins from various pathways were identified. However, without direct knowledge of the tolerance level of the different cultivars under the various stress treatments, it was difficult to make meaningful conclusions. 

### 2.3. Rice

#### 2.3.1. Drought

Several groups have studied the effects of drought or water deficit on the proteome in different cultivars of rice. Ji et al. [23] used quantitative proteomics to investigate flag leaf proteins in two rice cultivars: Zhenshan97B, a lowland drought-susceptible variety and the upland, drought-tolerant cultivar IRAT109. The sensitive variety showed a general decrease in protein abundance (15 decreased and 2 increased), while the tolerant variety showed the opposite trend with 14 proteins showing an increased abundance and only 6 proteins showing a decrease in abundance. Among the increased proteins in IRAT109 were the antioxidant enzymes, dehydroascorbate reductase, and superoxide dismutase. In only a single case did the responsive proteins correspond to the same protein in both cultivars; this was an ATP synthase beta subunit which was decreased in Zhenshan but increased in IRAT109.

Another rice proteomic study compared protein abundance changes in three rice cultivars differing in drought-tolerance; NSG19, a tolerant variety, Thai jasmine rice KDML105, of intermediate tolerance, and IR20 a sensitive cultivar. In this study polyethylene glycol (PEG 6000) was used to elicit the water stress response [25]. Analysis of drought-responsive proteins identified 53 (8.5% of total) that showed significant differences in abundance between the three cultivars. Enrichment for responsive proteins involved in cell and DNA repair were observed in the tolerant line, as well as an increase in the amount of coronatine-insensitive 1 protein, shown to have a role in stomatal closing, which may help to limit evaporative water loss and thus confer drought-tolerance. 

#### 2.3.2. Salinity

Salekdeh et al. [24] studied salt-responsive proteins in total protein extracts from roots of the tolerant rice cultivar Pokkali and the sensitive rice cultivar IR29 following long-term exposure. Analysis of 2D gels identified two proteins which were upregulated exclusively in the tolerant cultivar under stress conditions, including an abscisic acid stress ripening protein, ASR1, and a protein involved in lignin synthesis, caffeoyl-CoA O-methyltransferase. They also identified higher constitutive levels of the ROS scavenger ascorbate peroxidase in the salt-tolerant cultivar, but this protein was upregulated in both cultivars under salinity stress. 

#### 2.3.3. Nitrogen Deficiency

Nitrogen-deficiency stress responsive proteins in two rice cultivars differing in nitrogen use efficiency (Chunyou 58—tolerant and Yongyou 6—sensitive) was studied by a proteomics approach using 2DGE [26]. Gel analysis detected 31 protein spots in the two cultivars that showed reproducible changes, with four of these spots stress-inducible in both of the varieties. These shared proteins included ribulose-1, 5-bisphosphate carboxylase/oxygenase activase, Rubisco large subunit, glycine decarboxylase, as well as a transposase. In Chunyou 58, only 10 proteins showed changes in abundance while in the sensitive cultivar Yongyou 6, 27 proteins were altered, with many of these being identified previously as nitrogen deficiency-related proteins, including heat shock protein GSTF14, fibrillin-like protein, glutathione S-transferase and DegP2 protease.

### 2.4. Soybean

#### 2.4.1. Aluminium

Duressa et al. [27] carried out a quantitative proteomic analysis using 2D-DIGE of soybean root total protein under aluminium stress to compare the response of an aluminium tolerant (PI416937) to a sensitive cultivar (Young). The aluminium tolerant cultivar showed a more rapid protein response to the onset of the stress, with more proteins responding at a shorter exposure time, than the sensitive cultivar. Up-regulated proteins in the tolerant cultivar included malate dehydrogenase, enolase, malate oxidoreductase and pyruvate dehydrogenase. These enzymes contribute to the increased synthesis of organic acids, in particular citrate, which has been measured in this cultivar and shown to be secreted and important for binding and detoxifying Al [27]. A number of proteins involved in stress protection were also specifically enhanced in PI416937, and included thioredoxin, dehydroascorbate reductase as antioxidants and cysteine synthase and isoflavone reductase which contribute to Al detoxification. 

#### 2.4.2. UV-Irradiation

Quantitative proteomics was employed by Xu et al. [28] to study the impact of solar ultraviolet-B (UV-B) radiation on the soybean leaf proteome to investigate the protective role of flavonoids against UV-B, using two isolines which differ in their flavonoid content; a standard line that had moderate levels and magenta line that had reduced flavonoid levels making it susceptible to UV-B. Results indicated that many more proteins were responsive to solar UV-B in the reduced flavonoid line (29 increased, 22 decreased) than in the standard line (8 increased, 14 decreased) and the majority of these were categorized as functioning in cellular energy reactions, including multiple protein spots for the oxygen evolving enhancer protein 1 (OEE) of photosystem II and subunits of PS1. Another 12 proteins were not altered by UV-B but showed different accumulation between the two lines including carbonic anhydrase, and GCV H-protein, which were both identified by two spots which showed higher abundance in the standard line. In general, the proteins belonged to diverse functional groups and there was no obvious pathway enrichment.

#### 2.4.3. Salt

Salinity tolerance of two soybean genotypes; Jackson (salt-sensitive) and Lee 68 (salt-tolerant), was investigated using a 2DGE quantitative proteomic approach of total proteins extracted from leaves of seedlings [29]. Analysis of protein abundance changes showed 91 protein spots out of a total of 800 reproducible spots were salt-responsive. Identification of 78 of these indicated enrichment in proteins involved in free radical scavenging in the salt-tolerant line, including ascorbate peroxidase, glutathione-S-transferase, ferritin light chain precursor, and protein disulphide isomerase.

### 2.5. Barley

#### 2.5.1. Drought

Kausar et al. [34] investigated the changes in the protein profile of shoot total protein from three-day-old barley seedlings from a drought-sensitive genotype 004186 and a drought-tolerant genotype 004223 subjected to short-term drought stress (both PEG and water withholding experiments). Interestingly, plants responded differently to water stress applied by withholding water compared to water stress from PEG treatment with only a few responsive proteins shared between these treatments (3 out of 35 in the sensitive genotype, and 8 out of 36 in the tolerant genotype), suggesting that PEG application is not necessarily comparative to drought by water withholding. Highest fold change in protein abundance in the sensitive cultivar was observed for a Ptr Tox-A binding protein 1 and a 50 S ribosomal protein L9. While in the tolerant cultivar protein spots for a methionine synthase and a photosystem 1 reaction centre subunit II showed the highest fold changes. Key differences between the drought-tolerant and sensitive cultivars were observed in metabolism, defense, and photosynthetic related proteins, which were increased in the tolerant cultivars and decreased in the sensitive cultivars.

In another study aimed at understanding barley drought-tolerance, Ashoub et al. [37] compared the response of shoot total proteins of two Egyptian barley land races, accession number 15141, which is drought-tolerant, and accession number 15163, considered drought-sensitive, using DIGE. While there were a number of drought-induced protein changes that were shared between cultivars including increases in methionine synthase, ATP synthase alpha subunit, aconitase, alanine glyoxylate aminotransferase, ATP-dependent CLp protease, HSP 90, and protein disulphide isomerase, there were also cultivar specific changes. In the tolerant cultivar, these included Elongation factor EF2, metalloprotease, HSP 70, and a stress responsive protein. In the sensitive cultivar leucine aminopeptidase, lipoxygenase, sucrose synthase, betaine aldehyde dehydrogenase and NADP-malic enzyme all were increased with no changes in the tolerant cultivar. However, as changes were relatively small and proteins belonged to a wide range of functional process groups, no clear picture emerged which would help explain the different responses of the two cultivars to drought stress.

#### 2.5.2. Drought and Heat

One of the only reports touching on the importance of studying the effects of combined stresses on crop plants was carried out by Rollins et al. [35], which used 2D-DIGE technology to study the molecular basis of stress responses in leaf total protein extracts following single or combined exposure to drought and heat treatments in two drought-adapted, genetically diverse genotypes; the Syrian landrace selection Arta and the Australian barley cultivar Keel. Keel tended to show higher yield under drought and heat alone, while Arta tended to show a higher yield under the combination treatment. From a total of 1005 protein spots, DIGE analysis identified 305 spots significantly differentially regulated by the heat treatment, and 473 spots different between genotypes, with 35 spots different due to the interaction between temperature and genotype. Surprisingly, no spots were found to be significantly regulated by the drought treatment despite the severity of the stress (soil water content was reduced to 15%). Only a small proportion of these stress-responsive proteins were identified by mass spectrometry. One protein, glyceraldehyde-3-phosphate dehydrogenase, was induced by heat, genotype and a combination of heat and genotype. A significant enrichment in proteins with roles in photosynthesis were differentially abundant between the genotypes and also responsive to temperature, including oxygen-evolving enhancer protein 1 and 2, and chlorophyll a/b binding protein. However, while these proteins increased in abundance, the photosynthetic efficiency showed a significant reduction [35].

#### 2.5.3. Salinity

The assessment of salt-stress responses between contrasting barley cultivars was carried out using 2DGE focusing on root total proteins using the barley genotypes Steptoe (salt-sensitive) and Morex (salt-tolerant) [30]. Gel analysis identified 760 protein spots of which 39 were differentially expressed with most showing a cultivar- rather than a stress-specific response and more proteins were affected by the stress in the sensitive genotype than in the tolerant genotype. According to functional annotation of the identified proteins, enrichment was observed for those involved in oxidative stress responses with eight proteins functioning in redox regulation. These included MDAR, ascorbate peroxidase, and lactoylglutathione lyase, which were selectively down-regulated by salinity in Morex, while ascorbate peroxidase was up-regulated in Steptoe, highlighting genotype differences. In this study, the validation of spot expression changes for four of the identified proteins was carried out by Western blot analysis and results indicated a good agreement between the two approaches.

In a similar study by the same group, Witzel et al. [31] expanded on the results summarized above by using a narrower pH range of the IPG strips employed for 2DGE and tracking the expression of proteins over multiple time points. From 685 matched spots they could detect 91 which were significantly differentially expressed with a fold-change of at least 2; a much larger number than in their previous study. Interestingly, in this study, a higher number of proteins were induced by salinity in the tolerant genotype, while in their previous study more proteins were responsive to stress in the sensitive genotype [30]. As identified previously [30], there was involvement of ROS scavenging proteins but also MEP pathway enzymes, including hydroxymethylbutenyl 4-diphosphate synthase and 4-diphosphocytidyl-2-C-methyl-D-erythritol kinase were induced in both cultivars. No direct comparisons were made between the two studies which would have been helpful.

Rasoulnia et al. [32] and Fatehi et al. [33], also used quantitative proteomics (2DGE) to investigate salinity responsive total proteins in different barley genotypes but in this case comparing leaf proteins from seedlings of the salt-tolerant Afzal cultivar to the salt-sensitive L.527 barley genotype exposed to both short term and long-term stress. Under short-term salinity stress, a total of 117 salt-responsive proteins were detected in the two genotypes, with the identity of 13 reported in the manuscript. In general, the tolerant genotype showed a higher number of positively responsive proteins while the salt-sensitive genotype showed a higher number of proteins with decreased abundance. Proteins that were increased in the salt-tolerant cultivar but decreased in the salt-sensitive cultivar included: phosphoribulokinase, sedoheptulose 1,7-bisphosphatase, plastocyanin photosystem I subunit VII proteins, oxygen-evolving enhancer protein 2, 2-cys peroxiredoxin, and glutathione-S-transferase. The fact that many of these proteins are chloroplast localized and involved in photosynthesis may suggest that differences in salinity tolerance between the cultivars are due to a reorganization of photosynthetic machinery.

Under long-term salinity stress a total of 44 salt-responsive proteins in the two genotypes were identified [33]. Interestingly, proteins that were specifically induced in the tolerant cultivar under short term stress were induced in both cultivars under long-term stress; including oxygen evolving enhancer protein. In addition, in contrast to the short-term stress, many photosynthetic and carbon assimilation related proteins were induced in both genotypes.

#### 2.5.4. Boron

Patterson et al. [36] used iTRAQ labelling to look for boron-induced differences in root soluble protein abundances between two barley lines that are defined by the presence or absence of both the 4H and 6H boron-tolerance loci, which encode an anion-permeable transporter and a member of the NIP aquaporin family, respectively [51,52]. Four proteins showed an increase in abundance in the B-tolerant plants. Three of these proteins are involved in production of phytosiderophores, and the fourth was a defence-related protein. All four proteins have previously been demonstrated to be increased in expression in response to iron deficiency [53], and may be indicators of a general mineral deficiency response.

### 2.6. Brassica

#### Low Phosphate

Comparative 2DGE analyses of two Brassica oilseed lines exposed to long-term phosphorus deficiency; genotype 102 which was low phosphorus (LP) tolerant and genotype 105 which was sensitive to LP, was carried out to investigate protein abundance changes in total protein extracted from both roots and leaves [38]. A total of 32 proteins in leaves and 43 proteins in roots of the two lines showed reproducible and significant changes under LP conditions. In general, the sensitive line showed a greater number of proteins which increased in abundance in both the roots and leaves compared to the tolerant line which showed the opposite trend. Of those proteins that were shared (3 in leaves and 2 in roots), differential regulation was observed for 1 of these in both tissues; TAU6, a tubulin alpha-6 chain protein in roots and formyltetrahydrofolate deformylase related transferase in leaves. Functional analysis of those proteins that increased in genotype 105 showed enrichment for proteins involved in signal transduction, gene transcription, secondary metabolism, and stress proteins. While those proteins that decreased in genotype 102 were enriched in proteins related to gene transcription, protein translation, carbon metabolism, and energy transfer. 

### 2.7. Sugar Beet

#### Drought

Hajheidari et al. [42] compared and contrasted the drought-induced changes in the proteome of sugar beet leaf total protein extracts from two genotypes (7112 and 7219-P69) that differ in their genetic background. From a total of 500 detected protein spots, 79 showed significant changes under drought. Results highlighted genotype-specific patterns of protein responses to drought, with the number and degree of downregulated proteins higher in genotype 7219-P.69 and a higher number of drought-specific proteins identified in 7112, despite little difference in the physiological response of the genotypes to drought. The identity of only 11 of these proteins was reported, with genotype differences in response observed in two, a GATA zinc finger family protein and the Rubisco large subunit. 

### 2.8. Peanut

#### Drought

Kottapalli et al. [39] used a quantitative proteomics approach to study leaf soluble protein extracts from peanut genotypes differing in water deficit response (COC041—tolerant and COC166—sensitive). The study identified 79 differentially expressed protein spots which corresponded to 48 proteins. Lipoxygenase and 1L-myo-inositol-1-phosphate synthase were more abundant in tolerant genotypes as was Acetyl-CoA carboxylase, a key enzyme in lipid biosynthesis. Additionally, there was a marked decrease in the abundance of several photosynthetic/carbon assimilation proteins in the tolerant genotype, including Rubisco small and large subunits, carbonic anhydrase, chlorophyll a/b binding protein, and oxygen evolving enhancer protein 2, along with a concomitant decrease in photosynthetic rate and transpiration in response to water-deficit stress. This may reflect an adaptive measure to reduce oxidative damage during times of stress.

### 2.9. Sugarcane

#### Drought

Two cultivars, the drought-tolerant cultivar K86-161 and the drought-susceptible Khon Kaen 1 cultivar, were studied to identify drought-responsive alterations in proteins from leaves following long-term water deficit [40]. Analysis of 2D-gels detected 128 protein spots which showed a response to water deficit in the two cultivars, however only a small proportion of these were identified by mass spectrometry. Two of the identified proteins were specific to the drought-tolerant cultivar K86-161 and corresponded to a sugarcane protease inhibitor and a homolog of replication protein A1. Two proteins were identified as stress-responsive in both cultivars; ATPase beta chain and Actin. Another nine of these proteins showed an increase in abundance under stress conditions in the drought-tolerant cultivar compared to the drought-sensitive cultivar, and included RuBisCO activase, ferredoxin and an unknown protein referred to as p18. Increased p18 abundance in the drought-tolerant cultivar was verified in both the original cultivars as well as four additional cultivars, two drought-tolerant and two drought-sensitive cultivars, using ELISA and Western blotting.

### 2.10. Strawberry

#### Cold 

A comprehensive analysis using both 2DGE and label free quantitative proteomics (LFQP) was employed to identify differentially expressed total proteins in strawberry crown tissue, after short (2 day) and long (42 day) exposure to cold treatment, from two cultivars which differed in their freezing tolerance; a cold-tolerant cultivar, Jonsok, and a cold-sensitive cultivar, Frida [41]. A total of 22 proteins were consistently more abundant in Jonsok than in Frida at all experimental conditions suggesting that the cold tolerant cultivar is primed to respond. Notably, one of these, a thaumatin-like glucanase, shown to protect against freezing, was 70-fold higher in Jonsok than Frida, constitutively, but also accumulated to over 6000 fold higher in Jonsok compared to Frida after 42 days of cold treatment. In general, the cold sensitive cultivar Frida showed enrichment in responsive proteins for those involved in flavonoid biosynthesis, while the freezing tolerant Jonsok was enriched in stress responsive proteins involved in antioxidation, detoxification and disease resistance. When comparing the two quantitative approaches following two-day stress, 22 proteins were identified in both 2DGE and LFQP approaches, however, eight of these showed differential regulation.

### 2.11. Tomato

#### Salinity

A proteomic analysis using 2DGE of two contrasting tomato genotypes (Roma—salt-tolerant and SuperMarmande—salt-sensitive) was employed to identify salt-responsive genotype specific proteins [43]. A total of 26 protein spots exhibited significant abundance differences between treatments with enrichment of proteins involved in oxidative stress, stress defence and heat shock, and photosynthesis. Many of these were consistently higher after salt treatment in the salt-tolerant cultivar Roma, including various peroxidases, as well as heat shock proteins, suggesting that this variation could be related to the degree of genotype salt tolerance.

### 2.12. Chickpea

#### Drought

In order to gain more understanding of the role of the nucleus in the drought stress response, Subba et al. [44] compared the nuclei-enriched proteome of a dehydration-sensitive chickpea cultivar (ICCV-2) with that of JG-62, a tolerant cultivar. Analysis revealed cultivar-specific differential expression of many proteins involved in various cellular functions. Results highlight the dehydration-induced expression of enzymes associated with ROS scavenging, exclusively in JG-62; including superoxide dismutase, ascorbate peroxidase and GSH peroxidase. Furthermore, several of the drought-responsive proteins upregulated in JG-62 were found to be downregulated in ICCV-2, and included glyoxalase, histone H2 and H3, as well as several small heat shock proteins (CaSN-641 and 654).

### 2.13. Grape

#### Drought and salinity

Vincent et al. [45] carried out a proteomic study of the response to drought and salinity stress of two *V. vinifera* cultivars, Chardonnay and Cabernet Sauvignon. Both varieties displayed physiological differences when exposed to water deficit and salinity, with Chardonnay appearing to be more tolerant to water deficit and salinity than Cabernet Sauvignon. While several proteins showed similar responsiveness to stress in both cultivars, including a nuclear matrix constituent protein NMCP1, varietal differences were the main source of protein expression variation. The enrichment of proteins involved in protein degradation in Cabernet Sauvignon that were increased in abundance under either stress was consistent with the observation that this grapevine cultivar was less able to cope physiologically with water and salt stresses. Other notable differences were the large decrease in a NAC related transcription factor and the increase in a mitochondrial peroxiredoxin in Cabernet Sauvignon, whereas they were not altered in Chardonnay. None of the antioxidant enzymes identified in their protein profiling analysis were modulated by the stress conditions in either cultivar.

### 2.14. Banana

#### Drought

Vanhove et al. [46] used a Musa biodiversity collection to investigate the effects of water limitation induced by sorbitol treatment on the proteome of five varieties of in vitro propagated banana showing differences in their growth response to the mild drought stress. DIGE analysis of leaves from untreated and treated explants showed approximately 8% of the proteins were differentially expressed and 24 of these were identified by MS/MS. Results showed increased levels of defense related proteins, proteins involved in ROS detoxification and additionally dehydrogenases involved in NAD/NADH homeostasis [46]. However, an HSP70 protein was the most highly altered protein in the drought stressed tissue and further analysis led to the identification of the cytoplasmic HSP70 as the chromosome 2 paralog [54].

## 3. Common Stress Responses

One of the main pathways that was consistently upregulated despite the specific stress in stress-tolerant cultivars from the numerous crops reported here are those for the cellular defence machinery involved in ROS scavenging. This is considered an early general stress response to the overproduction of reactive oxygen species, which include superoxide and hydroxyl radicals as well as hydrogen peroxide [55]. This response is aimed to limit the effects of oxidative stress which damages nucleic acids, oxidizes proteins, and causes lipid peroxidation. The responsiveness of ROS scavenging enzymes, like isoforms of superoxide dismutase, catalase, ascorbate peroxidase, and glutathione-s-transferase were noted in multiple studies.

Similarly, heat shock proteins were also highly represented in the studies regardless of the stress response. These proteins have been shown to play an important role in multiple biotic as well as abiotic stress responses including pathogen attack, wounding, heat, cold, drought, salinity and UV [56]. They are thought to provide protein quality control and homeostasis, being intricately involved in protein folding, subunit assembly, delivery and degradation [57]. Heat shock proteins help to stabilize proteins and membranes during times of stress so protein function is not compromised [56]. 

Another widely detected abiotic stress responsive protein is oxygen evolving enhancer protein, an auxiliary component of the photosystem II (PSII) cluster required for maximal efficiency of the water oxidation reaction [58]. It is thought that it may function to stabilize PSII under stress conditions by protecting the catalytic manganese cluster and maintain correct thylakoid membrane architecture [59]. 

The universal nature of these common responses across multiple stress factors and various species precludes these proteins as effective stress biomarkers. Of more interest for biomarker discovery are those proteins that show responsiveness under specifically defined conditions and are species- and stress-specific, with links to clear phenotypes in the cultivars/genotypes tested. The ability to mine the genetic diversity in specific crops will accelerate the identification of relevant biomarkers. These have been noted in particular cases in Section 2. 

## 4. Quantitative Approaches, Phenotyping and Technical Constraints

The majority of studies summarized here employ label-free 2DGE approaches (25 out of 34), with only a few employing DIGE technology (6) or gel-free approaches, including iTRAQ (4) or LFQP (1). The preference for 2DGE is a reflection of the fact that the technique is relatively economically more attractive than the other more technologically advanced approaches, requiring significantly less mass spectrometry hours when compared to complex shot-gun type proteomics techniques, and does not require any specialized equipment or complex data analysis [60]. Furthermore, quantitative analysis can be easily carried out on a large series of multiple biological replicates, although this necessitates the running of a considerable number of gels, which could increase the technical variability inherent in this approach. Nevertheless, the possibilities are well beyond the multiplexing capacities of mass spectrometry label-based quantification, which in the case of iTRAQ is limited to eight in commercial kits, although with hyperplexing able to expand this to 18 in special experimental cases using triplex metabolic labelling and six-plex isobaric tags [61]. Additionally, this technique is effective in identifying post-translational modifications and alternative splicing of proteins [62,63]. Despite these advantages, 2DGE is restrictive in its depth of analysis as it is able to resolve only a limited collection of highly abundant and mostly soluble proteins with hydrophobic proteins poorly represented [62]. Many of the proteins that are identified using the 2DGE technique appear to be shared between studies regardless of the stress, tissue or species. This is highlighted in cross view analysis of proteomic data from multiple species which compare differentially responsive proteins in humans, rats, mice worms, and even fruit fly which demonstrate that specific proteins were over-represented in 2DGE quantitative analysis studies including glycolytic enzymes, heat shock proteins, and ROS scavenging enzymes [64,65], including enolase, HSP70, elongation factors, and superoxide dismutase. Remarkably, this list matches many of the proteins identified in the plant studies reviewed here. This is not to say that these proteins are not valid stress sensors, however, to be used as biomarkers, candidate proteins should be specific to a particular stress condition [65,66]. To identify more specific biomarkers we need to go beyond identifying highly abundant proteins. This implies that there is a need to focus on low abundant proteins via fractionation of samples to reduce complexity, yet in the studies reviewed here only three out of a total of 30 used samples other than total protein extracts. These included soluble proteins isolated by pelleting membrane fractions [36], proteins from purified mitochondria [20], and nuclear proteins [44].

An important factor in quantitative proteomics analysis, which determines the robustness of the results, is the number of technical and biological replicates employed. This is an important aspect of the experimental design and contributes to the statistical power of the analysis [67]. A number of the studies reported in this review failed to specify the number of replicates, and several used pooled samples rather than replicates (Table 1). It remains the responsibility of editors and reviewers to ensure studies that are published are statistically sound.

Crop yield is determined by many factors throughout the plant cycle, and thus studies that encompass crop performance to various stress regimes over multiple time points provide a better alternative to single stress and time point measurements, which provide only a limited picture of a given plant stress process. Additionally, the correct phenotyping of the genotypes/cultivars is essential to confirm the tolerance or sensitivity of the lines prior to proteomics. This involves the application of accepted protocols to measure a specific trait related to plant structure or function and should be confirmed under the specific growth conditions and treatments of the study [68]. 

Of the 30 proteomics data sets examined, approximately two thirds (25) compared only one treatment condition or time point with one control condition or time point. Only a single study compared both multiple stress conditions with various time points, including both short and long-term stress [31]. The majority of the studies also involved analysis of proteins from plants at a very young developmental stage, which make it difficult to extrapolate the actual stress tolerance to that of the crops that are more dependent on longer term plant performance and cyclic stress conditions. 

Finally, essential to biomarker discovery is the ability to systematically validate the quantitative proteomics study as it depends on complex computational and statistical analysis that while attempting to limit false discovery rate cannot unequivocally exclude it [66]. Therefore, to ensure the robustness of the data and for meaningful biomarker discovery, independent testing is crucial; yet only six studies mentioned here attempt to validate their proteomic results (Table 1). The most common form of validation is Western blot analysis, and was used by Jangpromma et al. [40] to confirm changes in protein amount for an unknown protein, p18, in different genotypes under drought stress. Good correlation between 2DGE and Western blot analysis was also observed for increases in β-actin protein and decreases in histone H4 after UV-B irradiation in corn [14]. Kottapalli et al. [39], and Alvarez et al. [18], use PCR to confirm the change in abundance of select proteins in their studies. However, mRNA levels did not always correlate well with protein levels. Witzel et al. [30,31], also reported a high degree of correlation between 2DGE and Western blot analysis for salinity stress responsive proteins in barley, showing results for catalase [30], HMGB2, HDS, and FQR1 [31] proteins. 

## 5. Conclusion

Biomarker discovery is a long and difficult process, as witnessed by the fact that in spite of all the efforts in the past decade in human disease research only a handful of protein biomarkers have been taken from the discovery phase, through confirmation and validation steps, to be used in diagnostics [66]. However, plants offer an attractive advantage for biomarker discovery in that the natural diversity available within genotypes of major crop plants can be exploited to allow for the identification of specific traits responsive to a particular environmental condition, from within a large and diverse sample pool. This would allow for a more confident selection of stress tolerance factors. These approaches may not only ensure agricultural yield sustainability in the face of changing environmental conditions but might equally allow for the increased use of degraded or marginal lands for agricultural production or the conservation or rejuvenation of ecosystems. Crop varieties that yield more with fewer inputs on sub-optimal soils will be pivotal to success.

## Figures and Tables

**Table 1:** A list of published quantitative proteomics papers in the field of crop plant responses to abiotic stress factors which employ different genotypes of common crop cultivars. Columns indicate the crop plant, stress condition, genotypes compared, age of plant at end of stress treatment, duration of stress treatment, tissue studied, methods of quantitative proteomics used, number of biological replicates, type of protein sampling, and whether the proteomic results were further validated.

Crop	Stress	Genotypes	Plant Age	Stress Duration	Plant Tissue	Quant. Method ^a^	# Biol. Reps. ^b^	Protein Extraction	Val. ^c^	Ref.
**Corn**	Drought–dehydration	CE704–tolerant, 2023–resistant	40 days	6 days	Leaves	iTRAQ/2DGE	NS/3	total protein	No	[14]
	Drought–water deficit	Lc–sensitive, Io–tolerant	3 weeks old	4, 6, 8, 10, 12, 14 days	Leaves	2DGE	3 or 5	total protein	No	[15]
	UV-B irradiation	W23–sensitive, Cacahuacintle-tolerant, Confite Puneño–tolerant	28 days	21 days	Leaves	DIGE	3	total protein	Yes	[16]
**Wheat**	Drought	Excalibur-tolerant, RAC875-tolerant, Kukri-sensitive	adult plants unknown age	cyclic	Leaves	iTRAQ	1	total protein	No	[17]
	ABA	Nesser–tolerant, Opata–sensitive	10 day old seedlings	6 h	Roots	iTRAQ	3	total protein	Yes	[18]
	Drought	Hanxuan10–tolerant, Chinese Spring–sensitive	seedlings	48 h	roots, leaves and sections including leaf sheath and stem	2DGE	3	total protein	No	[19]
	Salt 200 mM	CS–sensitive, AMP–tolerant	7 weeks	7 weeks	Shoots/Roots	DIGE	3	mitochondria	No	[20]
	Drought, heat, salt, cold	China-108, Yennon-78, Norin-61, Kantou-107	4 months	N.S.	Seeds	2DGE	1	total protein	No	[21]
	Drought and salt	Shanrong 3–tolerant, Jinan 177–sensitive	seedlings	24 h	Shoots/Roots	2DGE	3	total protein	No	[22]
**Rice**	Drought	Zhenshan97B–susceptible, IRAT109–tolerant	50 days	20 days	Leaves	2DGE	NS	total protein	No	[23]
	Drought	IR62266–tolerant, CT9993–sensitive	up to 53 days	various	Leaves	2DGE	NS	total protein	No	[24]
	Drought	KDML105–unknown, NSG19–tolerant, IR20–sensitive	25 days	up to 96 h	Leaves	1DGE	3	total protein	No	[25]
	Salinity 100 mM	Pokkali–tolerant, IR29–sensitive	28 days	14 days	Roots	2DGE	3	total protein	No	[24]
	Nitrogen deficiency	Chunyou 58–tolerant Yongyou 6–sensitive	4th leaf	up to 7 days	Leaves	2DGE	3	total protein	No	[26]
**Soybean**	Aluminium	PI416937–tolerant, Young–sensitive	seedlings	up to 72 h	Roots	DIGE	NS	total protein	No	[27]
	UV-B irradiation	Clark–tolerant, Magenta–sensitive	12 days	9 days	Seedlings	2DGE	5	total protein	No	[28]
	Salinity 150 mM	Jackson–sensitive, Lee–tolerant	21 days	up to 144 h	Leaves	2DGE	3	total protein	No	[29]
**Barley**	Salinity 50–250 mM	Morex–tolerant, Steptoe–sensitive	seedlings	13 days	Roots	2DGE IPG 3–10	3	total protein	yes	[30]
	Salinity 100–150 mM	Morex–tolerant, Steptoe–sensitive	seedlings	up to 16 days	Roots	2DGE IPG 4–7	3	total protein	yes	[31]
	Salinity 300 mM	Afzal–tolerant, Line 527–sensitive	seedlings	4 days	Leaves	2DGE	3	total protein	No	[32]
	Salinity 300 mM	Afzal–tolerant, Line 527–sensitive	7 weeks	3 weeks	Leaves	2DGE	NS	total protein	No	[33]
	Drought	004223–tolerant, 004186–sensitive	6 day old seedlings	3 days	Shoots	2DGE	3	total protein	No	[34]
	Drought	Arta–tolerant, Keel–tolerant	33 days	7 days	Leaves	DIGE	3	total protein	No	[35]
	Boron	DH +-tolerant, DH-–sensitive	seedlings	2 weeks	Roots	iTRAQ	2	soluble protein	No	[36]
	Drought	15141–tolerant, 15163–sensitive	Seedlings–unknown age	7 days	Leaves	DIGE	3	total protein	No	[37]
**Brassica**	Low phosphorus	102–tolerant, 105–sensitive	41 days	26 days	Leaves/Roots	2DGE	3	total protein	No	[38]
**Peanut**	Drought–water deficit	COC041-tolerant, COC166-sensitive	74 days	7 days	Leaves	2DGE	3	total protein	Yes	[39]
**Sugar cane**	Drought	K86-161-tolerant, KhonKhan-sensitive	15 weeks	21 days	Leaves	2DGE	NS	total protein	Yes	[40]
**Strawberry**	Cold	Frida-sensitive, Jonsok-tolerant	8 weeks + hardening 2–48 days	48 h	Crowns	2DGE and LFQP	3	total protein	No	[41]
**Sugar beet**	Drought	7112 7219	157 days	N.S.	Leaves	2DGE	10	total protein	No	[42]
**Tomato**	Salinity 100 mM	Roma–tolerant, Supermarmande–sensitive	24 days	14 days	Leaves	2DGE	4	total protein	No	[43]
**Chickpea**	Drought	JG-62–tolerant, ICCV-2–sensitive	27 days	1–6 days	Shoots	2DGE	2	nuclei	No	[44]
**Grape**	Drought and Salinity (250 mM final)	Chardonnay–tolerant, Cabernet Sauvignon–sensitive	Two-year-old rooted cuttings	up to 16 days	Shoots	2DGE	3	total protein	No	[45]
**Banana**	Drought (sorbitol)	Mbwazirume Williams Popoulou Obino L’Ewai Cachaco	4 week old explants	48 days	Leaves	DIGE	6	total protein	No	[46]

^a^ Quantification method; ^b^ Number of biological replicates; ^c^ Validation; NS, Not stated in manuscript.
